# Isolation and molecular characterization of a major hemolymph serpin from the triatomine, *Panstrongylus megistus*

**DOI:** 10.1186/1756-3305-7-23

**Published:** 2014-01-14

**Authors:** Carlos JC Moreira, Peter J Waniek, Richard H Valente, Paulo C Carvalho, Jonas Perales, Denise Feder, Reinaldo B Geraldo, Helena C Castro, Patricia Azambuja, Norman A Ratcliffe, Cícero B Mello

**Affiliations:** 1 Laboratório de Doença Parasitárias, Instituto Oswaldo Cruz, Fundação, Oswaldo Cruz (Fiocruz), Av. Brasil 4365, Rio de Janeiro, RJ 21045-900, Brazil; 2 Laboratório de Bioquímica e Fisiologia de Insetos, Instituto Oswaldo Cruz, Fundação, Oswaldo Cruz (Fiocruz), Av. Brasil 4365, Rio de Janeiro, RJ 21045-900, Brazil; 3 Laboratory of Toxinology, Instituto Oswaldo Cruz, Fiocruz, Av. Brasil 4365, Rio de Janeiro, RJ 21040-900, Brazil; 4 Laboratory for Proteomics and Protein Engineering, Carlos Chagas Institute, FIOCRUZ, Curitiba 81350-010, Brazil; 5 Laboratório de Biologia de Insetos, Universidade Federal Fluminense, Morro do, Valonguinho s/n, Cx Postal 100436, Niterói, RJ CEP 24001-970, Brazil; 6 LABiEMol, GCM - IB, Universidade Federal Fluminense, Morro do, Valonguinho s/n, Cx Postal 100436, Niterói, RJ CEP 24001-970, Brazil; 7 Department of Biosciences, College of Science, Swansea University, Singleton Park, Swansea, SA28PPWales, UK

**Keywords:** *Panstrongylus megistus*, Serpin, Hemolymph, Serine protease, Cleavage sites, *T. cruzi* serpin modulation

## Abstract

**Background:**

Chagas disease kills 2.5 thousand people per year of 15 million persons infected in Latin America. The disease is caused by the protozoan, *Trypanosome cruzi*, and vectored by triatomine insects, including *Panstrongylus megistus*, an important vector in Brazil. Medicines treating Chagas disease have unpleasant side effects and may be ineffective, therefore, alternative control techniques are required. Knowledge of the *T. cruzi* interactions with the triatomine host needs extending and new targets/strategies for control identified. Serine and cysteine peptidases play vital roles in protozoan life cycles including invasion and entry of *T. cruzi* into host cells. Peptidase inhibitors are, therefore, promising targets for disease control.

**Methods:**

SDS PAGE and chromatograpy detected and isolated a *P. megistus* serpin which was peptide sequenced by mass spectrometry. A full amino acid sequence was obtained from the cDNA and compared with other insect serpins. Reverse transcription PCR analysis measured serpin transcripts of *P. megistus* tissues with and without *T. cruzi* infection. Serpin homology modeling used the Swiss Model and Swiss-PDB viewer programmes.

**Results:**

The *P. megistus* serpin (PMSRP1) has a *ca*. 40 kDa molecular mass with 404 amino acid residues. A reactive site loop contains a highly conserved hinge region but, based on sequence alignment, the normal cleavage site for serine proteases at P1-P1′ was translocated to the putative position P4′-P5′. A small peptide obtained corresponded to the C-terminal 40 amino acid region. The secondary structure of PMSRP1 indicated nine α-helices and three β-sheets, similar to other serpins. PMSRP1 transcripts occurred in all tested tissues but were highest in the fat body and hemocytes. Levels of mRNA encoding PMSRP1 were significantly modulated in the hemocytes and stomach by *T. cruzi* infection indicating a role for PMSRP1 in the parasite interactions with *P. megistus.*

**Conclusions:**

For the first time, a constitutively expressed serpin has been characterized from the hemolymph of a triatomine*.* This opens up new research avenues into the roles of serine peptidases in the *T. cruzi/P. megistus* association. Initial experiments indicate a role for PMSRP1 in *T. cruzi* interactions with *P. megistus* and will lead to further functional studies of this molecule.

## Background

In 1909, Carlos Chagas identified a new human disease and its infectious biological agent, *Trypanosoma cruzi*[1]. He described the parasite’s life cycle in a wild mammalian host as well as the insect vector, a hemipteran from the family Reduviidae, subfamily Triatominae [2] that was classified as *Panstrongylus megistus*. This species is highly adaptable to a variety of ecosystems and has now become one of the most important vectors of Chagas disease in Brazil [3].

Subsequently, descriptions of more than 140 species of triatomines, distributed in 15–19 genera have been reported [4-6] but the genera most studied, due to their association with Chagas’ disease transmission, are *Triatoma, Panstrongylus, Rhodnius* and *Dipetalogaster*[7]. The biochemical and physiological similarities and differences of these vectors require further detailed research in order to more fully understand the factors responsible for the specificity occurring in various trypanosome/triatomine interactions [8].

Triatomines are exclusively hematophagous, hemimetabolic insects, passing through five nymphal instars before emerging as adults [9,10]. One triatomine vector species, *Rhodnius prolixus*, is easily raised in the laboratory and also usually molts following each blood meal so that these characteristics have contributed to this insect becoming a model for physiological studies [9].

In 2005, the National Human Genome Research Institute (NHGRI) designated *R. prolixus* as an important organism for genome sequencing [11]. The knowledge of the insect physiology accruing from such genome information could be used to identify targets for inhibiting the vectorial competence of triatomines and consequently to control Chagas disease [12]. Some genes related with triatomine immunity have already been identified [13] including defensin [14-16], lysozyme [14,17,18], prolixicin [19] and components of the Rel/Nuclear Factor kappa B family [20]. However, in triatomines, despite these latter studies, our knowledge of the regulation of physiological pathways, such as those controlled by the protease cascades that activate reproduction, development and immunity is strictly limited. In insect immunity, the prophenoloxidase system and recognition of pathogen-associated molecular patterns (PAMPs) involve protease cascades which are triggered when the host recognition receptors bind to PAMPs, including peptidoglycans, lipophosphoglycans or 1,3-beta-glucans [21-25].

Protease inhibitors generally regulate all protease cascades with serpins being the largest and most widely studied family of such inhibitors [23]. Serpins are found in both prokaryotes and eukaryotes and are involved in many biological processes, including the regulation of innate immune reactions in insects [22,24,25]. Serpins are typically composed of 350–400 amino acid residues and contain an exposed reactive center loop (RCL) which binds to the active protease site [26,27]. Serpins can adopt different conformational states and can either be active and stressed (native form) or inactive and relaxed (latent form). The native state is unstable with the RCL exposed and poised to interact with the target protease. After interaction, there is cleavage of the scissile bond (P1-P1′) and the RCL becomes linked to the protease covalently and, assisted by the breach and shutter regions, inserted into the β-sheet A to stabilize the structure (e.g. [28]). Serpins in their latent states can also be cleaved but remain stable and inactive. When highly concentrated, serpins can acquire inactive polymeric structures [27,29,30].

Many sequences of insect serpins have been deposited in public databases [24], but in triatomines, as far as we are aware, this kind of protease inhibitor has only been described in a truncated sequence of a contig from the cDNA library of the *Triatoma infestans* sialome [31].

Although there is a lack of information on serpins in triatomine/*T. cruzi* associations, a role for these molecules in other insect vector/parasite interactions has already been established. Thus, in mosquito/*Plasmodium* and tsetse fly/African trypanosomes interactions with several serine proteases and their inhibitory serpins have been described, and details of their roles in controlling the Toll and prophenoloxidase immune activation pathways are emerging [32,33]. The important role of serpins in the life cycle of *T. cruzi* is, however, implied by the detection of multiple serine protease genes in this flagellate [34] and by the role of serine proteases in the invasive stage of the parasites in the mammalian phase of the life cycle. Thus, the propyl oligopeptidase family of serine proteinases (oligopeptidase B and Tc-80) has been shown to be involved in parasite adhesion and entry into host cells [35]. More recently Oliveira *et al*. [36,37] described the presence of heparin binding proteins (HPBs) on the surface of all stages of *T. cruzi* which modulate the attachment of the parasites to glycosaminoglycans on both mammalian and insect cells. These HPBs were shown to be localized at the flagellar membrane and, subsequently, to have serine protease activity [36,37]. Such serine proteases are usually associated with complex cascades that amplify signals and are controlled by serine protease inhibitors [24].

The present paper derives from an initial comparative appraisal of the hemolymph protein profiles of vector insects from the genera *Triatoma*, *Panstrongylus*, *Rhodnius* and *Dipetalogaster* that led to the discovery of a major serpin in the hemolymph of *P. megistus*. Here, we describe the purification and characterization of this serpin as well as a putative role for this molecule in the interaction of *T. cruzi* with its *P. megistus* host. The identification of such a potentially important host factor could assist manipulation of the vector physiology to block *T. cruzi* development or even to compromise the ability of the vector insect to resist disease.

## Methods

### Ethics statement

The animals used to maintain the insects at FIOCRUZ were treated according to the Ethical Principles in Animal Experimentation approved by the Ethics Committee in Animal Experimentation (CEUA/FIOCRUZ) approved under the protocol numbers P-54/10-4/LW12/11 and L-0061/08. Both protocols are from CONCEA/MCT (http://www.cobea.org.br/), which is associated with the American Association for Animal Science (AAAS), Federation of European Laboratory Animal Science Associations (FELASA), International Council for Animal Science (ICLAS) and Association for Assessment and Accreditation of Laboratory Animal Care International (AAALAC).

### Insects and hemolymph collection

The insects used in the experiments were fifth instar nymphs of the following species: *Dipetalogaster maximus, Triatoma infestans, P. megistus, Panstrongylus lutzi, R. prolixus, Rhodnius neglectus* and *Rhodnius brethesi* from colonies established and maintained at Laboratório de Doenças Parasitárias, FIOCRUZ, as described by Carvalho-Moreira *et al*. [38]. The insects were fed on anesthetized chickens until the 3th instar and thereafter fourth instar nymphs received citrated rabbit blood, from CECAL-FIOCRUZ-RJ, using an artificial apparatus [39].

Hemolymph was collected from fifth instar nymphs, 5 days after feeding from insects anesthetized on ice and carefully cleaned with 70% ethanol, by excising the metathoracic legs and gently pressing the abdomen. Drops of hemolymph were collected with micropipettes and pooled in Eppendorf tubes, on ice, containing a few crystals of phenylthiourea (Sigma-Aldrich, St. Louis, MO, USA) to prevent melanization [40]. The hemolymph was then centrifuged at 5000 × *g* for 5 min and the supernatants stored at −20°C until use.

### Electrophoresis of hemolymph samples

SDS-PAGE (14% to 16%) was performed on a Mini-Protean II system (Bio-Rad, Hercules, CA, USA) under reducing conditions, using 4% stacking gels [41]. Staining was carried out with colloidal Coomassie Brilliant Blue [42] or with silver nitrate [43]. To compare the profiles of the hemolymph protein of triatomines, 0.1 μl hemolymph samples from each species were used. The molecular mobilities of proteins were determined by interpolation from mobilities of commercial pre-stained standards (Sigma-Aldrich) by computer analysis. Proteins from the hemolymph and samples from chromatography were quantified with a protein test kit (Sigma-Aldrich) using bovine serum albumin (BSA) standards [44].

### Purification of *P. megistus* hemolymph serpin

Anion exchange chromatography was carried out with 2 ml of Q-Sepharose Fast Flow (Sigma-Aldrich) in Poly-Prep plastic columns (Bio-Rad, USA) at 4°C. The column was washed and the sample (1 ml of hemolymph) was equilibrated with 0.01 M phosphate buffer (pH 7.2). The chromatography proceeded in step gradients with 8 buffer elution samples of 40 ml each. The first step was eluted with 0.01 M phosphate buffer and the subsequent steps by the addition of 0.05 M; 0.075 M; 0.1 M; 0.125 M; 0.15 M; 0.2 M and 0.3 M of NaCl in the phosphate buffer. The fractions from anion exchange were filtered in Centricon 100 (Millipore, Billerica, MA, USA) and thereafter dialyzed against H_2_O and concentrated using a centrifugal filter Centriprep 30 (Millipore) at 4°C. The more enriched samples with the target protein were used for gel digestion with trypsin and mass spectrometric analyses (see below).

For preliminary activity assays, enriched samples were also submitted to low pressure molecular exclusion chromatography with Sephacryl 100 in a 1.0 × 100 cm, glass Econo-Column (Bio-Rad), at 4°C. The column was washed and eluted with PBS (0.14 M NaCl in 0.01 M phosphate buffer, pH 7.2, 380 mOsm) and equilibrated with Cytochrome C and vitamin B12 standards. The column was loaded with 0.5 ml of sample, and 0.75 ml fractions collected after 25 ml of discarded void volume. Protein concentrations of fractions were determined and the protein profiles analyzed by SDS-PAGE, as described above.

### Trypsin digestion of selected SDS-PAGE bands

**Figure 1:**
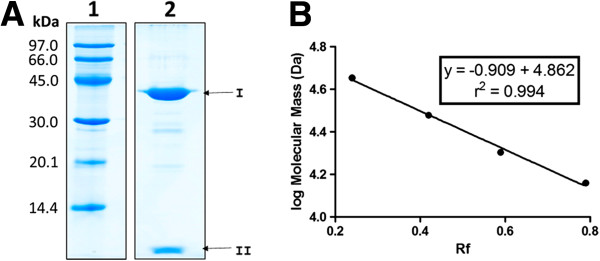
**Relative molecular mass determination of *P. megistus *hemolymph fraction (PMSRP1).** PMSRP1 fraction was purified by anion exchange chromatography/Centriprep 30 concentration. **A**- 15% SDS-PAGE under reducing conditions. Lane 1; molecular mass markers: phosphorylase b (97 kDa), albumin (66 kDa), ovalbumin (45 kDa), carbonic anhydrase (30 kDa), trypsin inhibitor (20.1 kDa) and α-lactalbumin (14.4 kDa); Lane 2; *P. megistus* target protein fraction at 38.8 kDa (arrow I) and low mass band (arrow II). **B**- Optimized calibration curve used for relative molecular mass determination.

The ca. 40 kDa band (Figure 1A-I) and the low molecular mass (less than 10 kDa) band (Figure 1A-II) detected by SDS-PAGE analysis of the protein purified in the previous step were submitted to tryptic digestion [45]. The eluted peptides were completely dried on a SpeedVac concentrator (Thermo Scientific, Waltham, MA, USA), resuspended in 12 μl of a 1% (v/v) formic acid solution and kept at −20°C until mass spectrometric analyses, described below.

### Peptide sequencing by high resolution mass spectrometry

The tryptic peptide digests were submitted to reversed-phase nanochromatography coupled to nanoelectrospray high resolution mass spectrometry for identification. Peptides were eluted online in a LTQ XL/Orbitrap mass spectrometer (Thermo Scientific) for analysis. MS1 spectra were acquired on the Orbitrap analyzer (300 to 1,700 *m/z*) at a 60,000 resolution. For each spectrum, the 10 most intense ions were submitted to HCD (higher energy collisional dissociation) followed by MS2 acquisition on the Orbitrap analyzer at 15,000 resolution.

The raw data files generated from the duplicate mass spectrometric analyses were submitted to PEAKS version 6.0 *build* 20120620 (Bioinformatics Solutions Inc., Canada) for protein identification [46-49]. Peptide-spectrum matching (PSM) was performed against the non-redundant FASTA database of the National Center for Biotechnology Information (NCBInr, downloaded on September 4th, 2012) using Metazoa as taxonomical restriction and data were filtered for a 1% FDR at the peptide level. The mass spectra that did not yield any PSM according to *Peaks DB* but had high scoring *de novo* results were submitted to a similarity-driven search against the full NCBInr database using an in-house tool called PepExplorer. This tool is currently under development in the Laboratory for Proteomics and Protein Engineering (ICC-FIOCRUZ, Brazil). Briefly, it relies on the Smith-Waterman algorithm [50], the Peaks ALC *de novo* scores and machine learning to compose a final identification list.

After the complete sequence of the protein, described in the present article, was obtained by molecular biology, it was added to the full Swiss-Prot database (downloaded on November 15th, 2012) and the PEAKS analysis was repeated against this new database, maintaining all the other parameters as previously stated.

### Sequencing of *P. megistus* serpin (PMSRP1) cDNA

For the identification of PMSRP1 encoding cDNA, midgut and fat body of five *P. megistus* fifth instar nymphs at seven days after feeding were dissected. Total RNA was isolated using the NuceloSpin RNA II Kit (Macherey-Nagel, Düren, Germany), according to the manufacturer’s protocol. First strand cDNA was synthesized using the 3′-RACE Kit (Invitrogen, Karlsruhe, Germany), following the manufacturer’s instructions. For the subsequent PCR, the degenerate oligonucleotide primer PMSRP-F1 (5′-TGGGCNACNCARTTYAAYCC-3′), based on the amino acid sequence WATQFNP obtained by mass spectrometry and the reverse abridged universal amplification primer were used. The PCR fragments obtained were cloned into the pGEM-T Easy vector (Promega, Madison, WI, USA). Each clone was sequenced at least three times in both directions by Plataforma Genômica, Sequenciamento de DNA/PDTIS-FIOCRUZ, Rio de Janeiro, Brazil. Based on this first sequence, the oligonucleotides PMSRP-GSP1 (5′-CAGGTAAACCTGTAAT-3′), PMSRP-GSP2 (5′-CTTCATTTGAATATGGAAGCTCT-3′) and PMSRP-GSP3 (5′-GGAAGGTGGTTAAATGGAAATA-3′) were designed for the amplification of the 5′-end using a 5′ RACE Kit Version 2.0 (Invitrogen) according to the manufacturer’s instructions. The resulting amplicons were cloned and sequenced, as described above.

### Sequence analysis of PMSRP1

Sequence identity of the PMSRP1 encoding cDNA and comparison with the GenBank database was assessed using the BLASTX program at the web servers of the National Center for Biotechnology Information (http://www.ncbi.nlm.nih.gov/) [51]. Derived serpin amino acid sequences were aligned using ClustalW version 2.1 and slightly manually corrected [52]. Putative signal peptide cleavage sites were calculated with SignalP Version 4.0 [53]. The predicted isoelectric point and molecular mass was determined with the Compute pI/MW tool at http://expasy.org/tools. The secondary structure was predicted using J-pred program (http://www.compbio.dundee.ac.uk/~www-jpred/).

### Reverse transcription (RT)-PCR analysis of *P. megistus* tissues

Total RNA was extracted, using the NucleoSpin RNAII Kit (Macherey-Nagel), from salivary glands (SG), stomach (ST, = crop or anterior midgut), small intestine (SI, = posterior midgut), fat body (FB) and hemocytes (HC) of *P. megistus* fifth instar nymphs (n = 10) at 7 days after feeding with heat-decomplemented rabbit blood containing 2 × 10^6^ cells/ml *T. cruzi* strain Dm28c. Control insects were fed on blood without parasites. *P. megistus* gDNA was extracted from stomach tissue of five insects using the Wizard SV Genomic DNA Purification Kit (Promega). Prior to dissection, insects were immersed in water at 55°C for 15 s to detach hemocytes from other tissues [54]. First-strand cDNA was synthesized from 1–3 μg total RNA using the First-Strand cDNA Synthesis Kit (GE Healthcare, Buckinghamshire, UK) according to the manufacturer’s protocol. To verify that no genomic DNA remained, the gene encoding *T. brasiliensis* defensin 1 (*def1*), which contains an intron of 107 bp, was initially amplified as a cDNA control [14,16].

PCRs were carried out to detect relative levels of serpin cDNAs using the specific primers PMSRP-RT-F (5′-GAATTGCTGAGAATTTGTATGC-3′) and PMSRP-RT-R (5′-ATGTTGAAGAACTTTAAACATTG-3′) at the following cycling conditions: 94°C for 25 s; 52°C for 25 s; 72°C for 30 s and resulted in amplicons of 301 bp. The cycle numbers (25, 30 and 35) were empirically optimized to exclude signal saturation. PCRs were undertaken three times under the same conditions using technical replicates. For an internal control and standardization, the gene encoding ß-actin was amplified, as described previously [55,56]. As negative controls, PCR reactions were carried out lacking a template. Amplification products (5 μl) were separated on an ethidium bromide stained 2% agarose gel and documented with an EDAS 290 gel documentation system (Kodak, Rochester, NY, USA). Band intensity was measured with the ImageJ program (version 1.47v). Means and standard deviations of the different samples were calculated. One-way ANOVA and Student’s *t*-tests were carried out to evaluate significant differences in the various tissues and between infected and non-infected insects. All nucleic acid experiments were performed on a Veriti 96-Well Fast Thermal Cycler (Applied Biosystems, Carlsbad, CA, USA). For verification of primer specificity all obtained PMSRP1 amplificates were purified and sequenced as described above.

### Construction of the PMSRP1 model

Initially, the homology model of serpin was constructed as described by Abreu *et al*. [57], using the Swiss Model and Swiss-PDB viewer programs available at http://swissmodel.expasy.org/andhttp://www.expasy.org/spdbv/, respectively [58,59]. The set of structurally conserved regions (SCRs) was constructed based on the crystal structure of the serpin from *Tenebrio molitor* (PDB entry code 3OZQ). *T. molitor* serpin structure (1.9 Å crystal resolution) did not have a reactive center loop (RCL) that was built based on serpin B3 (PDB = 2ZV6) with a root mean square deviation (RMSD) of 1.34 Å. Blocks of structurally conserved regions were identified and the structure alignment of the serpin sequences was generated. Coordinates for all residues were transferred to the serpin sequence and loops were constructed in a single round. Several cycles of constrained energy minimization regularized the structures and their geometrical parameters. In subsequent runs, the serpin model was minimized and validated [60]. The prediction of the electrostatic potential map (MEP) was also performed in the Swiss PDB viewer program. It was generated in the range from 25.0 (deepest red color) to +30.0 (deepest blue color) kcal/mol and superimposed onto a molecular surface of constant electron density of 0.002 e/au3. Each point of the three dimensional molecular surface map expresses the electrostatic interaction energy value evaluated with a probe atom of positive unitary charge, providing an indication of the overall molecular size and location of attractive (negative) or repulsive (positive) electrostatic potentials shown in red and blue, respectively.

## Results

### Hemolymph protein profile and pre-purification

**Figure 2:**
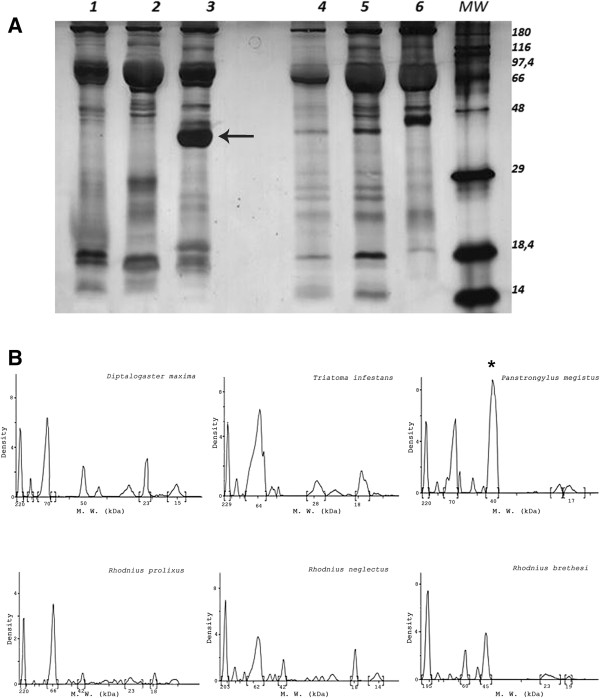
**Hemolymph supernatant protein profiles from triatomine vector species and graphics analysis of the bands. A**- Samples (0.1 μL of each supernatant) were submitted to SDS-PAGE (16%) analysis. *Dipetalogaster maximus* (lane 1)*, Triatoma infestans* (lane 2)*, Panstrongylus megistus* (lane 3)*, Rhodnius prolixus* (lane 4)*, Rhodnius neglectus* (lane 5) and *Rhodnius brethesi* (lane 6). MW - molecular mass markers. Arrow indicates the target protein at ca. 40 kDa. Figure represents a single gel containing all 6 hemolymph samples run simultaneously. **B**- Graphics analysis of the bands corresponding to the hemolymph protein profiles in SDS-PAGE. The x axis quantifies in arbitrary units the density of each protein band and the y axis the molecular mass derived from the molecular mass markers. *P. megistus major target protein of ca. 40 kDa.

In the present study, a comparative appraisal of the hemolymph protein profiles of triatomine vector species was initially undertaken in order to identify potential hemolymph factors that could be responsible for variations in triatomine vector competence to transmit *T. cruzi and Trypanosoma rangeli*[13]. The results showed that the protein profiles from the hemolymph of *D. maximus, T. infestans, P. megistus, R. prolixus, R. neglectus* and *R. brethesi* had similar banding patterns in the SDS PAGE gels above 60–70 kDa (Figure 2). However, only in the *P. megistus* hemolymph was there a major band with a molecular mass of ca. 40 kDa (Figure 2). The other species also had bands around 40–50 kDa, but none of them represented a major hemolymph protein. Subsequently, a similar band of ca. 40 kDa was also detected in the hemolymph from another *Panstrongylus* species, *P. lutzi* (Additional file 1: Figure S1).

The initial purification of the *P. megistus* ca. 40 kDa hemolymph protein by anion exchange chromatography showed that the fraction which eluted with 0.125 M NaCl was the most highly enriched for the ca. 40 kDa target protein and was selected for subsequent analysis. Under an optimized calibration curve using Rf values for molecular mass standards, only from 14.4 to 45 kDa, in 15% reducing SDS-PAGE (Figure 1A), the molecular mass of the band was recalculated as 38.8 kDa (Figure 1B). This mass is smaller than that predicted for the full length protein by the molecular biology data as 43.1 kDa (see below). A possible explanation for this discrepancy is provided by the presence of a less intense, small molecular mass band smaller than 10 kDa at the bottom of the gel (Figure 1A-II). Subsequent mass spectrometric analysis of this band confirmed that it corresponded to the cleaved C-terminal region of PMSRP1 (see below).

### Mass spectrometric analyses

**Figure 3:**
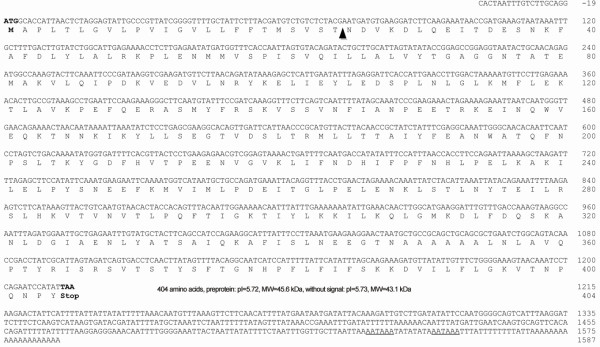
**Nucleotide sequence of PMSRP1 encoding cDNA and its deduced amino acids sequence.** Initial and terminal codons are in bold and the two polyadenylation signals are underlined. The black arrow indicates the putative signal peptide cleavage site after Thr24.

The 40 kDa band was excised from SDS-PAGE gels and digested with trypsin followed by analysis with reversed phase nanochromatography coupled online to high resolution mass spectrometry. The generated data were analyzed with PEAKS 6.0 software using an algorithm that combines *de novo* sequencing, peptide sequence tag and peptide-spectrum match (PSM) against an NCBInr database restricted to Metazoa. Unfortunately, no protein was identified other than the common keratin contaminants and trypsin autolysis products. The results produced 1,372 mass spectra that did not yield any PSM according to Peaks DB but had high scoring *de novo* results. These *de novo* results were therefore submitted to a similarity-driven search against the full NCBInr database using an in-house tool called PepExplorer. This approach identified the major protein present in the SDS-PAGE band as a member of the SERPIN (*P. megistus* serine proteinase inhibitor, designated PMSRP1) family (Table 1). The first seven amino acids of one of the peptides (i.e., WATQFNPSLTK) were chosen for designing primers for further molecular biology assessments (Additional file 2: Figure S2). The annotated *de novo* sequence for the full peptide is shown in Figure 3, which illustrates the initial and terminal codons and the two polyadenylation signals.

**Table 1:** **List of high-confidence *de novo *** **sequenced peptides (PEAKS 6.0 software) along with the sequence hits obtained when running the PepExplorer tool against the NCBInr full database**

	***PEAKS 6.0***		***PepExplorer***		**GI number**	**Protein description**	**Organism**
	**Sequence**^**a**^	**De novo score (%)**	**Sequence hit**	**Alignment score (%)**			
**1**	FNTQFNPSJTK	75	DSQFNPSLTK	60	3608131	Putative serpin	*Arabidopsis thaliana*
**2**	FJANPEETRK	91	FLANPEEARK	66	189029926	Putative serpin-Z6A	*Oryza sativa*
**3**	KJJEJPYSNEEFK	80	KVLELPYQNQEF	64	195120283	Serpin-like protein	*Drosophila mojavensis*
**4**	JVMMJPDEJTGJPEJENK	84	LLVMLPDDISGLAQLENK	81	224045098	Serpin, clade B, member 3	*Taeniopygia guttata*
**5**	JPDEJTGJPEJENK	93	LPDDISGLAQLENK	66	224045098	Serpin, clade B, member 3	*Taeniopygia guttata*
**6**	VMJMJPDEJTGJPEJENK	82	MLPDDISGLAQLENK	77	224045098	Serpin, clade B, member 3	*Taeniopygia guttata*
**7**	QFNPSJTK	78	QFNPSLTK	58	3608131	Putative serpin	*Arabidopsis thaliana*
**8**	VSSVDFJANPEEKTR	83	VDFLANPEE	67	189029926	Putative serpin-Z6A	*Oryza sativa*
**9**	VSSVDFJANPEETR	98	VDFLANPEEAR	74	189029926	Putative serpin-Z6A	*Oryza sativa*
	VSSVNFJANPEETR	97		68			
	VSDVNFJANPEETR	95		68			
	GKSVSSVNFJANPEETR	83		68			
	KSVSSVNFJANPEETR	81		68			
	VSDVDFJANPEETR	81		74			
	KSNVSSVNFJANPEETR	79		68			
	KNSVSSVNFJANPEETR	76		68			
**10**	VSSVNFJANPEETRK	91	VDFLANPEEARK	75	189029926	Putative serpin-Z6A	*Oryza sativa*
	SSVNFJANPEETRK	89		75			
	KGSVDFJANPEETRK	88		81			
	VSSVDFJANPEETRK	87		81			
	SVNFJANPEETRK	83		75			
	SKGVSSVNFJANPEETRK	77		75			
	SKGGVSSVNFJANPEETRK	75		75			
**11**	YPVMJPDEJTGJPELENK	83	VMLPDDISGLAQLENK	84	224045098	Serpin, clade B, member 3	*Taeniopygia guttata*
**12**	WATEFNPSJTK	87	WDSQFNPSLTK	61	3608131	Putative serpin	*Arabidopsis thaliana*
	WATQFNPSJTK	82		68			
	EANWATQFNPSJTK	80		68			

^a^ L or I equals J.

All sequences matched to members of the SERPIN family.

### Sequencing of PMSRP1 cDNA

**Figure 4:**
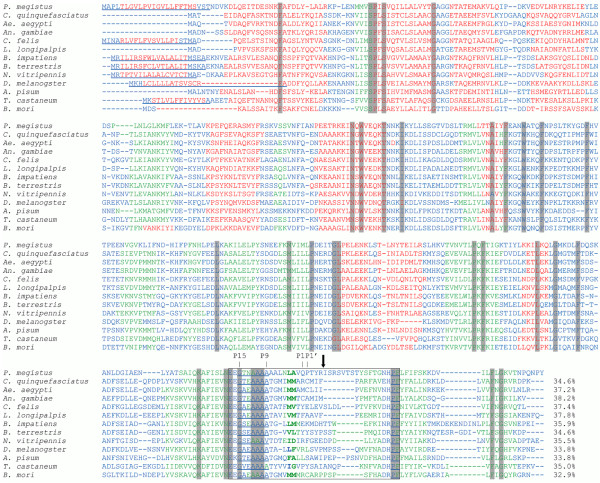
**Alignment of deduced amino acid sequence of *P. megistus *serpin (PMSRP1) compared with different insect serpins.** Identical amino acid residues between PMSRP1 and other serpins are shaded grey. The conserved reactive centre loop is boxed, the hinge regions and the PF residues of the “shutter region” are underlined. The putative proteolytic cleavage site, predicted by the alignment, is indicated by P1/P1′. Predicted serine proteases cleavage sites are marked in bold typeface. The true P1-P1′ of PMSRP1 (putative P4′-P5′) scissile bond is indicated by an arrow. The secondary structure was predicted by using the Jnet prediction method (http://www.compbio.dundee.ac.uk/~www-jpred/submit.html), ß-strands are highlighted in green, α-helices in red and the extended region in blue. The GenBank accession numbers for the analysed sequences are: *Culex quinquefasciatus* (XP 001865071), *Aedes aegypti* (XP 001658641), *Anopheles gambiae* (XP 314158), *Ctenocephalides felis* (AAN73325), *Lutzomyia longipalpis* (ABV60345), *Bombus impatiens* (XP 003487908), *Bombus terrestris* (XP 003399187), *Nasonia vitripennis* (XP 001606111), *Drosophila melanogaster* (CAB 63097). *Acyrthosiphon pisum* (BAH 71022), *Tribolium castaneum* (EFA 09186) and *Bombyx mori* (NP 001037021).

**Figure 5:**
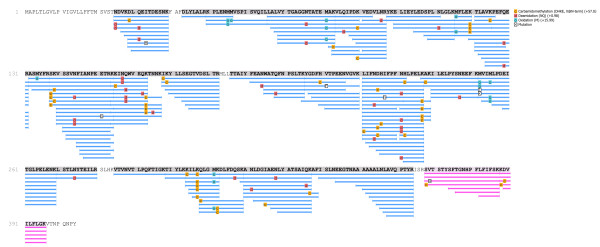
**Protein sequence coverage by mass spectrometry of the serpin purified from the hemolymph of *Panstrongylus megistus*.** Residues 1 to 24 correspond to the predicted signal peptide. Blue lines correspond to the digested peptides identified from band I and pink lines designate the peptides from band II shown in Figure 1A, relevant to the C-terminal region (residues 365 to 404).

After 3′ RACE with a degenerate forward primer 5′ RACE, and assembly of the sequences, PMSRP1 encoding cDNA (GenBank accession no. JX894893) of 1606 bp was identified using total RNA that came from the fat body of *P. megistus*. PMSRP1 comprised a 19 bp 5′ non-coding region and a 317 bp 3′ non-coding region between the stop codon (TAA) and the first polyadenylation signal (AATAAA). An open reading frame of 1212 bp encoded a deduced pre-protein of 404 amino acid residues with a predicted molecular weight of 45.7 kDa. At the PMSRP1 amino-terminus a putative signal peptide of 24 amino acid residues and a cleavage site after Thr were identified (Figure 3). The amino acid sequence NDVKDLQEITDESNK detected by mass spectrometry and the absence of sequences derived from the signal peptide supported the SignalP predicted amino-terminal end of mature PMSRP1 (Figures 3, 4 and 5). The theoretical molecular mass and isoelectric point of the mature protein were 43.1 kDa and 5.73, respectively (Figure 4).

The comparative analysis of the primary sequence alignment of the thirteen serpins from different insects showed that at position 344–381 of PMSRP1 there was a reactive center loop (RCL) which is a motif characteristic for serpins. The putative RCL based in the alignment includes the highly conserved flexible hinge region (GTNAAAA) at putative position P15-P9 (G[T/S]X[A/G/S]_4_) [61], the putative cleavage site (putative P1-P1′), and the PF residues of the “shutter region” (Figure 4). In addition, unlike most similar serpin amino acid sequences from other insects, Glu at putative P13 is substituted by an Asn, but like the serpins from the other insect species, the putative P8-P4′ region is highly variable (Figure 4). Finally, at putative positions P1 and P1′, based on sequence alignment, no cleavage site for serine proteases is present but chymotrypsin (Tyr) and trypsin (Arg) cleavage sites are present at putative P3′ and P4′, respectively (Figure 4). Thus, the putative position P4′- P5′ based on sequence alignment is the true scissile bond, P1-P1′, of PMSRP1 (Figures 4 and 5).

Mature PMSRP1 showed a low level of identity when compared with other serpin sequences available in the GenBank. The protein showed the greatest similarity of 38.2% with the sequences of *An. gambiae* (XP_314158) and a slightly lower of 37.8% and 37.4% with the sequences of *L. longipalpis* (ABV60345) and *C. felis* (AAN73325), respectively (Figure 4). Despite this level of identity, the theoretical prediction of the serpin secondary structure pointed to the conservation of nine α-helices and three β-sheets, similar to other serpin family members [61,62] (Figure 4).

After the full-sequence of the serpin present in the hemolymph of *P. megistus* was determined by deduction of mRNA sequence analyses, it was added to the full Swiss-Prot database and all the data were run using the PEAKS 6.0 workflow. The PMSRP1 was unambiguously identified with a sequence coverage of 87% (Figure 5). An important finding was that a 40-residue-long C-terminal region did not yield any peptide that matched its sequence, although tryptic sites (Arg and Lys) were present. Mass spectrometric analysis of the small band detected in the SDS-PAGE (Figure 1A-II) confirmed that it corresponded to the cleaved C-terminal region of PMSRP1 (Figure 5) undetected in the amino acid sequence obtained from the band with 38.8 kDa (Figure 1A-I). This supports the concept that the scissile bond, P1-P1′, occurs between the putative P4′ and P5′ (Arg-Ile) positions based on sequence alignment (Figures 4 and 5).

### 3D-Structure of PMSRP1

**Figure 6:**
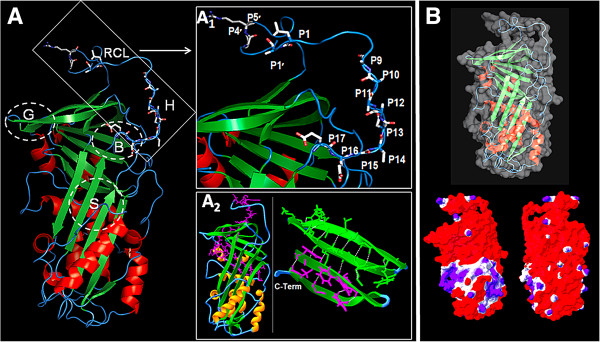
**Theoretical model of the PMSRP1. A**- The structure shows three β-sheets groups (green), nine α-helices (red), the breach (B), shutter (S), gate (G), hinge (H) and the predicted reactive center loop (RCL) regions [61,62]. A_1_ shows RCL and the putative residues P17-P9 and P1, P1′, P3′ and P4′ based on alignments. A_2_ left shows the 40-residue-long C-terminal region (pink) inserted in the structure, and A_2_ right shows C-terminal interacting with several H-bonds (white dots) helping to maintain a group of β-sheets. **B**- The serpin molecular surface (top) and the electrostatic potential maps (bottom) showing the distribution of positive (blue) and negative (red) regions on the molecular surface (left front and right back views).

In this study, we also constructed the PMSRP1 theoretical model using the *T. molitor* serpin and the serpin B3 crystal structures as templates (Additional file 3: Figure S3). The comparison of the PMSRP1 model with the templates revealed a highly conserved structure at both secondary (three β-sheets and nine α-helices) and three-dimensional levels (RMSD = 1.34 and 0.49 Å, serpin B3 and *T. molitor* serpin, respectively) (Figures 4 and 6). The conservation of the core of the structure reinforced the hypothesis of some authors that the serpin scaffold is intolerant to the deletion of all but peripheral elements of secondary structure [61,62]. Thus changes in non-conserved residues that allowed the folding of the serpin into an active native state were favored by the selective pressure.

Similar to other serpins, such as α-antitrypsin, breach, shutter, gate and hinge regions were identified in the PMSRP1 model (Figure 6). In addition, the 40-residue-long C-terminal region is involved in several H-bonds that help in organizing a group of α-sheets in the core of the protein (Figure 6). These H-bonds may help in retaining the C-terminal region in the structure in case of cleavage of the RCL, which is exposed in the serpin model. The potential electrostatic map of PMSRP1 revealed a large negative surface with some positive patches included on the reactive center loop (RCL) corresponding to residues Arg340 and Arg343 (Figure 6).

### PMSRP1 transcript abundance in different tissues

**Figure 7:**
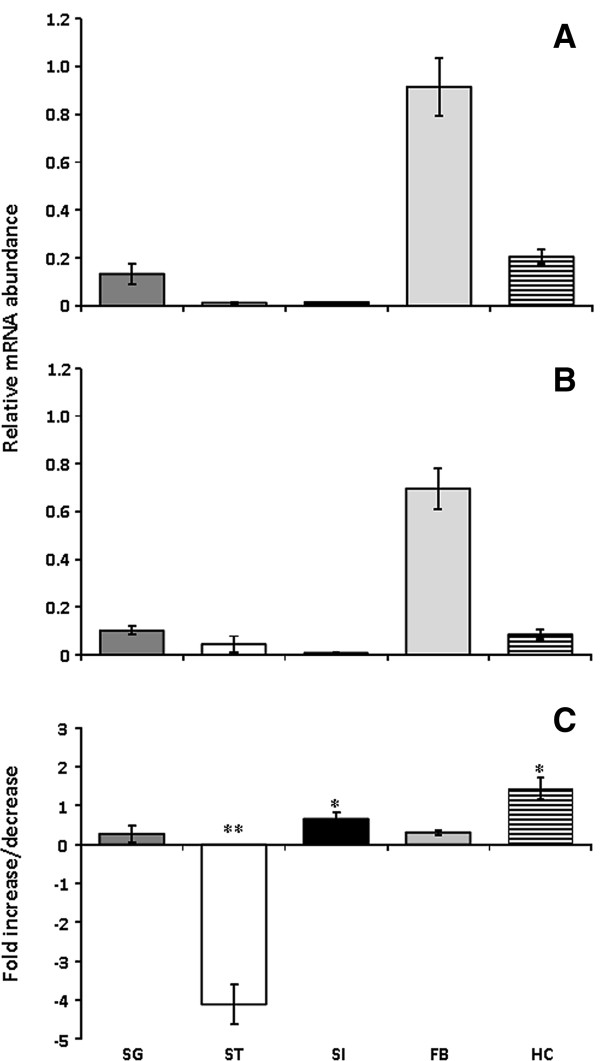
**Relative transcript abundance of PMSRP1 encoding mRNA in different tissues of fifth-instar nymphs after 25 cycles in relation to *ß-actin*, A- Seven days after a blood meal containing *T. cruzi *strain Dm28c (2 × 10**^**6 **^**cells/ml), B- Seven days after a parasite-free blood meal (control), C- Fold increase (positive axis) and decrease (negative axis) transcript abundance of *PMSRP1 *in infected insects (A) compared with the uninfected control (B).** Asterisks relates to significant differences (*P < 0.01, **P < 0.001) obtained after statistical analyses using one way ANOVA and Student’s *t*-test. SG – salivary glands, ST – stomach, SI– small intestine, FB – fat body, HC – hemocytes.

Reverse transcription PCR relative to *ß-actin* was used to measure *PMSRP1* transcript abundance in different tissues of fifth instar nymphs at seven days after feeding with blood containing *T. cruzi* or a parasite free meal (Figure 7). Preliminary amplification of the *P. megistus* defensin gene showed a single band of about 130 bp in all tissues analyzed and the absence of a 240 bp gDNA band (not shown). Therefore, it was assumed that there was no contamination of the mRNA with nucleic DNA. In negative controls, lacking cDNA and carried out for each RT-PCR, no amplification products were detected. After 30 and 35 cycles most PCR products were saturated and, therefore, band intensity of the PCR products after 25 cycles was quantified. PMSRP1 encoding mRNA was abundant in all tested tissues, in both *T. cruzi* infected and control insects, although in significantly different quantities (Figure 7). Graphical representation reflected the amplicon distribution in the gels of control insects (FB > SG/HC > ST > SI). In the stomach and small intestine, PMSRP1 transcript levels were lowest after 25 cycles. In insects given a *T. cruzi* infected blood meal 7 days previously, the *PMSRP1* transcript abundance decreased significantly in the stomach (*P* < 0.001) and increased in the small intestine (*P* < 0.01) (Figure 7C). In the salivary glands, the PMSRP1 transcript abundance was slightly higher than in the intestinal tract but did not differ significantly between infected and uninfected insects. In the hemocytes of control insects, the PMSRP1 transcript abundance was comparable with that in the salivary glands, but, following *T. cruzi* infection, the PMSRP1 abundance increased significantly in these cells (*P* < 0.01). The highest PMSRP1 levels were detected in the fat body of the insects but showed no significant differences following infection (Figure 7).

## Discussion

Although the triatomines acting as vectors of Chagas disease belong to the same subfamily, Triatominae, the members have unique anatomical, genetic, physiological and ecological characteristics [63]. Morphological differences between the species have been described and are used as taxonomic markers [4]. Such anatomical divergences are often associated with physiological variations specific for each species and occur despite the highly conserved chromosome number in triatomines. Karyotype comparisons between species show that there is cytogenetic variability in the sex chromosome number, chromosomal position of rDNA clusters, genome size and heterochromatin organization [5,64]. These genetic variations result in differences between species in the biochemical composition of the saliva [65,66], in the gut microbiota [67], in the feeding behavior, and in the colonization of specific ecotopes [68]. Variation in profiles of the hemolymph proteins between different species of triatomines is another characteristic resulting from these genetic divergences, as shown in the present paper (Figure 2). Such factors might influence the epidemiology of Chagas disease [69], and could assist in the reconstruction of the evolutionary history of the triatomines [70].

In the present paper too, although there were some similarities in the hemolymph banding patterns in the SDS PAGE gels of the different triatomine species, only in the *Panstrongylus spp.* was there a major band with a molecular mass of ca. 40 kDa, corresponding to the serpin, PMSRP1. This difference cannot be explained without further investigation of both the level of this band at various stages in the life cycles of triatomines, as well as the possible functions of this serpin in *P. megistus.* If the results reveal increases in *P. megistus* hemolymph serine protease levels prior to molting and decreases in PMSRP1 during molting then this could indicate a role for PMSRP1 in the regulation of cuticle degradation as in *Choristoneura fumiferana*[71]. Previously, the molting fluids of insects have been shown to contain several proteases, including a trypsin-like protease in *M. sexta,* probably involved in cuticle breakdown [72,73]. Following molting, these enzymes would need inhibition to protect the newly formed cuticle. This latter hypothesis has gained support from research with the serpins of the spruce budworm, *C. fumiferana*[71]. Since *P. megistus,* is a particularly long-lived insect with a highly sclerotized and melanized exoskeleton then degradation and shedding of the cuticle may present a particular problem, requiring higher levels of both proteases and associated serpin inhibition.

In addition, the vectorial competence between triatomine species is also highly variable with differences in the multiplication and development of *T. cruzi*[74] and *T. rangeli* in the insect hosts [75]. Variability of parasite development in these insects is also promoted by the specificity of parasite strains and clones for each vector species [38,76], which may reflect the differential efficacy of the triatomine immune response in each species.

In *P. megistus*, the major protein band in the hemolymph, identified by SDS-PAGE, was a serpin. A similar band was also present in *P. lutzi* hemolymph, confirming that the genus *Panstrongylus* has some homogeneity [77] although considerable variation in salivary gland proteins has been shown even within *P. megistus* populations [78]. Recently, a salivary gland serine protease has been described in *P. megistus*[79,80], as well as Kazal-type and lipocalin inhibitors. The Kazal-type inhibitor, however, was expressed in the stomach but not in the small intestine or hemocytes [81]. Nevertheless, work on *P megistus* is in its infancy with only 45 ESTs reported recently as available for this species [64] so that the detection of many new factors, such as the serpin in the stomach, fat body and hemocytes of the present paper, is to be expected. The fact that serpins have not been reported from other triatomine species, except for only a truncated cDNA cluster in the sialome of *Triatoma infestans*[31], may reflect the more recent evolution of this vector to the hematophagous habit and the need to adapt to changes in diet [78].

The amino acid sequence alignment of PMSRP1 with serpins from other insects indicates a low level of identity from 32.9 to 38.2% (Figure 4). Such heterogeneity among serpins is not unusual and can even occur within the same insect species as with the six serpins of *Manduca sexta* which have less than 40% amino acid sequence identity [82].

Nevertheless, the comparison of the sequence of PMSRP1 with those from other insects revealed several important common features such as the conservation in the reactive center loop (RCL) (Figures 4 and 6). Significantly, inhibitory serpins are characteristically recognized by a consensus sequence in their hinge with P17 (E), P16 (E/K/R), P15 (G), P14 (T/S), P12-P9 (A/G/S) [61,83]. In PMSRP1, the hinge sequence is in putative positions P17 (E), P16 (E), P15 (G), P14 (T), and P12-P9 (AAAA) which exactly matches the inhibitory sequence above. Non-inhibitory serpins would deviate from this sequence, be unable to effectively insert the RCL into the β-sheet A and therefore insert the RCL into an adjacent molecule to form non-inhibitory polymers with alternative functions (reviewed in [61,84]).

In addition, compared to most similar serpin amino acid sequences from other insects, Glu at P13 is substituted by Asn (Figure 4). Although it involves a charge change from the negative Glu to the uncharged Asn, according to our electrostatic potential map of the serpin model (Figure 6), there is no significant contribution of this substitution to the serpin charge distribution. This substitution may not be crucial since Glu and Asn are structurally similar, without extensive side-groups, and it has been shown previously in the hinge region that substitutions to uncharged residues have little effect [85].

PMSRP1, and all serpins from the other insect species, also have highly variable P8-P4′ regions (Figure 4) and include the predicted proteolytic cleavage sites P1 and P1′ which are known to contribute to the inhibitor specificity of serpins [86]. However, no cleavage site for serine proteases is present at the putative P1 or P1′ sites of PMSRP1 but chymotrypsin and trypsin cleavage sites, respectively, occur at Tyr and Arg of putative positions P3′ and P4′, respectively (Figure 4). Consequently, the P1-P1′ scissile bond of PMSRP1 is translocated to the putative position P4′-P5′ based on sequence alignment (Figures 4 and 5). The same amino acids, Arg-Ile, form the cleavage site for *Manduca sexta* serpins 4 and 5 as well as for 25 hemolymph serine proteases in this insect [87]. The serpin from the beetle *Sphenophorus levis* also shows an unusual cleavage site at Arg-Ile of the putative P2-P1 of the RCL with a fragment released from the C-terminus of 4.3 kDa that was not detected in SDS-PAGE analysis [88].

The *M. sexta* serpins 4 and 5 and the *S. levis* serpin are cleaved at Arg-Ile and probably involved in regulating prophenoloxidase (proPO) [82,89]. However, the *M. sexta* serpins do not inhibit the usual prophenoloxidase activating proteases (PAPs) but act upstream on hemolymph protease-6 to control both melanization and antimicrobial peptide expression [87]. Since PMSRP1 is also cleaved at Arg-Ile by trypsin and has the conserved hinge inhibitory consensus sequence, but not the usual proPO cleavage sequence, Asn-Lys-Phe-Gly, then PMSRP1 may still be involved in regulating pro-PO but upstream from the usual PAPs as with the *M. sexta* serpins.

An important finding was that a 40-residue-long C-terminal region of PMSRP1 did not yield any peptide that matched its sequence, although tryptic cleavage sites were present. The molecular mass of PMSRP1 was calculated to be 38.8 kDa, a significantly smaller value than that predicted as 43.1 kDa for the mature protein by the molecular biology data. A possible explanation for this discrepancy might be that the C-terminal region has a deduced molecular mass of 4.6 kDa. If this molecular mass is subtracted from the predicted molecular mass of the mature protein (43.1 kDa), the resulting new theoretical mass for the protein cut from the denaturing electrophoresis gel would be 38.5 kDa, in accordance with the experimental mass (38.8 kDa) determined by SDS-PAGE. This 40 amino acid residue peptide was cut at Arg-Ile of P1-P1′ (putative P4′-P5′) and removed from the C-terminus (Figures 4 and 5), as was confirmed by the amino acid sequence shown in Figure 5.

It is not unusual for cleavage in the RCL of a serpin to result in the release of a small C-terminal fragment as with PMSRP1. Thus, the plant serpin, WSZ3, with a molecular mass of ca. 42 kDa, can be cleaved in the RCL with various proteinases to release ca. 4 kDa C-terminal fragments and form another fragment of ca. 39 kDa [90]. These results corroborate those found for PMSRP1, since the SDS-PAGE shown in Figure 1A displays a low molecular mass band. As PMSRP1 was concentrated and dialyzed in Centriprep 30 (kDa), prior to SDS-PAGE, any small free peptides should have been discarded during the centrifugation. Therefore, the small polypeptide visualized at the bottom of the gel (Figure 1A-II), probably originated from the native PMSRP1 molecule during sample processing for electrophoresis, which is carried out under denaturing and reducing conditions. These conditions would break non-covalent and covalent bonds (such as disulfide bridges) between molecules.

The gene encoding PMSRP1 is expressed in hemocytes, fat body, salivary glands and digestive tube and, as shown by SDS-PAGE, the respective protein is present constitutively, at least in the hemolymph. Preliminary experiments with PMSRP1 failed to detect any protease inhibitory activity or any effect on hemocytes phagocytosis and prophenoloxidase activation (data not shown). This may be due to inactivation resulting from previous cleavage of the RCL to yield the less than 10 kDa C-terminal. Serpins in their native (active) forms are not that thermodynamically stable and can readily convert to their more stable, latent (inactive) conformation [91]. This conversion may result from interaction with extracellular matrix proteins, hemolymph metalloproteinases, or simply by dialysis, storage or freeze-thawing so that functional testing may require freshly purified protein at room temperature [92].

Extensive attempts failed to obtain the active form of PMSRP1 for detailed functional assays, utilizing insects injected with anticoagulants such as EDTA or the immunosuppressant, dexamethasone, or by recombinant technology (unpublished data). However, measuring mRNA levels in tissues of *P.megistus* following *T. cruzi* infection was more successful in initial experiments. The results showed that at 7 days post-infection with *T.cruzi*, PMSRP1 expression is significantly upregulated in the small intestine and hemocytes and downregulated in the stomach of insects. Previously it has also been shown in other insects that serpins involved in immunity are modulated by infection [89,93].

Interpretation of these results for modulation of PMSRP1 following *T. cruzi* infection refers to several previous studies on changes in expression of triatomine immune molecules and immune reactions in *R. prolixus* following *T. cruzi or Trypanosoma rangeli* parasitization. These studies include results with assays for hemocyte microaggregation, nitric oxide (NO), nitric oxide synthase (NOS) and prophenoloxidase (PpO) activities [54,94-96]. Unfortunately, variations both in insect sampling times post-infection and the use of both biochemical and molecular biology analyses in these studies complicate matters, although generalizations can be made. Thus, all these previous studies show, like the present results with PMSRP1, that both *T. cruzi* and *T. rangeli* can manipulate the host immune response to optimize their survival. For example, oral infection with *T. cruzi* enhances levels of PpO and antibacterial factors but reduces NO in the *R. prolixus* stomach to kill competing bacteria and aid parasite survival [94]. In the present study, the decrease in PMSRP1 expression in the stomach of *P. megistus* could potentially mediate such an increase in PpO levels in response to *T. cruzi* infection by controlling negatively the activity of proteases involved in triggering this process (e.g. [89]). In contrast, the increase in *PMSRP1* expression in the hemocytes recorded in *P. megistus* is also relevant since oral infection of *R. prolixus* with a non-invasive strain of *T. rangeli*, inhibited levels of both PpO and microaggregation reactions in the hemocoel [54,95-97]. An increased level of hemocyte PMSRP1 could well be responsible for such a down-regulation of the host immune responses.

## Conclusions

In conclusion, a serpin has been identified and characterized, for the first time in a triatomine, from the hemolymph of *P. megistus* and shown to be constitutively present. This finding is particularly important as it should open up new avenues of research into the roles of serine proteases in the association of *T. cruzi* with its insect vector host. Initial experiments indicate a role for the PMSRP1 in *T. cruzi* interactions with *P. megistus* but further studies are required to detail the functions of this molecule in vector insect/parasite interactions.

## Competing interests

The authors declare that they have no competing interests.

## Authors’ contributions

CJCM, PJW, RHV, HCC, PA, NAR, CBM designed the study protocols and drafted the manuscript; CJCM, DF, NAR and CBM carried out the experiments with insects and serpin pre-purification; CJCM, PJW and PA performed the molecular experiments; RHV, PCC and JP developed the biochemistry proteomic experiments; RBG and HCC undertook the protein modeling; All authors analyzed, interpreted the data, revised the article for intellectual content, approved of the version to be published and are the guarantors of the paper.

## Supplementary Material

Additional file 1: Figure S1 Hemolymph supernatant protein profile from *Panstrongylus lutzi*. Samples (0.1 μl of each supernatant) were submitted to 14% SDS-PAGE analysis. *Panstrongylus megistus* (lane 1)*, Panstrongylus lutzi* (lane 2)*, Dipetalogaster maximus* (lane 3)*, Rhodnius neglectus* (lane 4) and *Rhodnius brethesi* (lane 5). MW - molecular mass markers. Arrows indicate the target protein at ca. 40 kDa. Click here for file

Additional file 2: Figure S2 Fully annotated spectrum for peptide sequence WATQFNPSLTK after *de novo* sequencing using PEAKS 6.0 software. A precursor mass accuracy of 1.3 ppm and an ALC score of 82% were assigned to this peptide. Data were generated by high resolution acquisitions (Orbitrap analyzer) in MS1 and MS2 modes; fragmentation was performed by HCD (higher energy collisional dissociation) fragmentation. Click here for file

Additional file 3: Figure S3 Alignment of the primary sequence of *T. molitor and P. megistus* serpins. This alignment was used to construct the 3D model of PMSRP1 shown in Figure 6. The identical amino acids residues are represented by an asterisk (*), whereas residues with similar chemical properties and score > 0.5 in the Gonnet PAM250 matrix are (:) and those with different chemical properties and low-score ≤ 0.5 are (.). Click here for file

## References

[B1] Chagas C Nova tripanossomíase humana. Estudos sobre a morfologia e o ciclo evolutivo do *Schizotrypanum cruzi*, agente etiológico de nova entidade mórbida do homem Mem Inst Oswaldo Cruz 1909 1 159 218 10.1590/S0074-02761909000200008

[B2] Lent H Wygodzinsky P Revision of the Triatominae (Hemiptera: Reduviidae), and their significance as vectors of Chagas’ disease Bull Am Mus Nat Hist 1979 163 123 520

[B3] Barbosa SE Diotaiuti L Soares RP Periera MH Differences in saliva composition among three Brazilian populations of Panstrongylus megistus (Hemipteran, Reduviidae) Acta Trop 1999 72 91 98 10.1016/S0001-706X(98)00073-4 9924964

[B4] Galvão C Carcavallo R Rocha DS Jurberg J A checklist of the current valid species of the subfamily Triatominae Jeannel, 1919 (Hemiptera: Reduviidae) and their geographical distribution with nomenclatural and taxonomic notes Zootaxa 2003 202 1 36

[B5] Panzera F Pérez R Panzera Y Ferrandis I Ferreiro MJ Calleros L Cytogenetics and genome evolution in the subfamily Triatominae (Hemiptera, Reduviidae) Cytogenet Genome Res 2010 128 77 87 10.1159/000298824 20407223

[B6] Schofield CJ Galvão C Classification, evolution, and species groups within the Triatominae Acta Trop 2009 110 88 100 10.1016/j.actatropica.2009.01.010 19385053

[B7] Cruz-Lopez L Malo EA Rojas JC Morgan ED Chemical ecology of triatomine bugs: vectors of Chagas disease Med Vet Entomol 2001 15 351 357 10.1046/j.0269-283x.2001.00340.x 11776453

[B8] Garcia ES Azambuja P Development and interactions of *Trypanosoma cruzi* within the insect vector Parasitol Today 1991 7 240 244 10.1016/0169-4758(91)90237-I 15463507

[B9] Wigglesworth VB The Principles of Insect Physiology 1972 7 London: Chapman and Hall

[B10] Alves CL Araujo RN Gontijo NF Pereira MH Importance and physiological effects of hemolymphagy in triatomines (Hemiptera: Reduviidae) J Med Entomol 2011 48 372 381 10.1603/ME10151 21485376

[B11] Huebner E The *Rhodnius* Genome Project: The promises and challenges it affords in our understanding of reduviid biology and their role in Chagas’ transmission [abstract] Comp Biochem Physiol 2007 148 S130 S130

[B12] Boulanger N Bulet P Lowenberger L Antimicrobial peptides in the interactions between insects and flagellate parasites Trends Parasitol 2006 22 262 268 10.1016/j.pt.2006.04.003 16635587

[B13] Ursic-Bedoya R Lowenberger CA *Rhodnius prolixus*: identification of immune-related genes up-regulated in response to pathogens and parasites using suppressive subtractive hybridization Dev Comp Immunol 2007 31 109 120 10.1016/j.dci.2006.05.008 16824597

[B14] Araújo CA Waniek PJ Stock P Mayer C Jansen AM Schaub GA Sequence characterization and expression patterns of defensin and lysozyme encoding genes from the gut of the reduviid bug *Triatoma brasiliensis* Insect Biochem Mol Biol 2006 36 547 560 10.1016/j.ibmb.2006.04.003 16835020

[B15] Lopez L Morales G Ursic R Wolff M Lowenberger C Isolation and characterization of a novel insect defensin from *Rhodnius prolixus*, a vector of Chagas disease Insect Biochem Mol Biol 2003 33 439 447 10.1016/S0965-1748(03)00008-0 12650692

[B16] Waniek PJ Castro HC Sathler PC Miceli L Jansen AM Araujo CAC *Two novel defensin-encoding genes of the Chagas disease vector* Triatoma brasiliensis *(Reduviidae, Triatominae): gene expression and peptide-structure modeling* J Insect Physiol 2009 55 840 848 10.1016/j.jinsphys.2009.05.015 19505471

[B17] Ursic-Bedoya R Nazzari H Cooper D Triana O Wolff M Lowenberger C Identification and characterization of two novel lysozymes from *Rhodnius prolixus*, a vector of Chagas disease J Insect Physiol 2008 54 593 603 10.1016/j.jinsphys.2007.12.009 18258253

[B18] Waniek PJ Mendonca-Lima L Menezes GB Jansen AM Araujo CAC Recombinant expression and characterization of a lysozyme from the midgut of *Triatoma brasiliensis* (Hemiptera, Reduviidae) in comparison with intestinal muramidase activity Physiol Entomol 2009 34 309 317 10.1111/j.1365-3032.2009.00691.x

[B19] Ursic-Bedoya R Buchhop J Joy JB Durvasula R Lowenberger C Prolixicin: a novel antimicrobial peptide isolated from *Rhodnius prolixus* with differential activity against bacteria and *Trypanosoma cruzi* Insect Mol Biol 2011 20 775 786 10.1111/j.1365-2583.2011.01107.x 21906194

[B20] Ursic-Bedoya R Buchhop J Lowenberger C Cloning and characterization of Dorsal homologues in the hemipteran *Rhodnius prolixus* Insect Mol Biol 2009 18 681 689 10.1111/j.1365-2583.2009.00909.x 19754745

[B21] Cerenius L Kawabata S Lee BL Nonaka M Söderhäll K Proteolytic cascades and their involvement in invertebrate immunity Trends Biochem Sci 2010 35 575 583 10.1016/j.tibs.2010.04.006 20541942

[B22] Kanost M Serine proteinase inhibitors in arthropod immunity Dev Comp Immunol 1999 23 291 301 10.1016/S0145-305X(99)00012-9 10426423

[B23] Olson ST Gettins PG Regulation of proteases by protein inhibitors of the serpin superfamily Prog Mol Biol Transl Sci 2011 99 185 240 21238937 10.1016/B978-0-12-385504-6.00005-1

[B24] Gulley MM Zhang X Michel K The role of serpins in mosquito immunology and physiology J Insect Physiol 2012 59 138 147 22960307 10.1016/j.jinsphys.2012.08.015 PMC3560325

[B25] Park SH Jiang R Piao S Zhang B Kim EH Kwon H-M Jin XL Lee BL Ha N-C Structural and functional characterization of a highly specific serpin in the insect innate immunity J Biol Chem 2011 286 1567 1575 10.1074/jbc.M110.144006 21047786 PMC3020765

[B26] Dunstone MA Whisstock JC Crystallography of serpins and serpin complexes Methods Enzymol 2011 501 63 87 22078531 10.1016/B978-0-12-385950-1.00005-5

[B27] Huntington JA Read RJ Carrell RW Structure of a serpin-protease complex shows inhibition by deformation Nature 2000 407 923 926 10.1038/35038119 11057674

[B28] Khan MS Singh P Azhar A Naseem A Rashid Q Kabir MA Jairajpuri MA Serpin inhibition mechanism: a delicate balance between native metastable state and polymerization J Amino Acids 2011 Article ID 606797, 10 pages doi:10.4061/2011/606797 10.4061/2011/606797 PMC3268027 22312466

[B29] Dolmer K Gettins PGW How the serpin α _1_-proteinase inhibitor folds J Biol Chem 2012 287 12425 12432 10.1074/jbc.M111.315465 22334651 PMC3320992

[B30] Tsutsui Y Cruz RD Wintrode PL Folding mechanism of the metastable serpin *α*_1_-antitrypsin Proc Natl Acad Sci USA 2012 109 4467 4472 10.1073/pnas.1109125109 22392975 PMC3311335

[B31] Assumpção TC Francischetti I Andersen M Schwarz JF Santana A Ribeiro JM An insight into the sialome of the blood-sucking bug *Triatoma infestans,* a vector of Chagas’ disease Insect Biochem Mol Biol 2008 38 213 232 10.1016/j.ibmb.2007.11.001 18207082 PMC2262853

[B32] Baton LA Garver L Xi Z Dimopoulus G Functional genomics studies on the innate immunity of disease vectors Insect Sci 2008 15 15 27 10.1111/j.1744-7917.2008.00184.x

[B33] Mwangi S Murungi E Jonas M Christoffels A Evolutionary genomics of *Glossina morsitans* immune-related CLIP domain serine proteases and serine protease inhibitors Infect Genet Evol 2011 11 740 745 10.1016/j.meegid.2010.10.006 21055483

[B34] McKerrow JH Rosenthal PH Swenerton R Doyle P Development of protease inhibitors for protozoan infections Curr Opin Infect Dis 2008 21 668 672 10.1097/QCO.0b013e328315cca9 18978536 PMC2732359

[B35] Vermelho AB Nogueira de Melo AC Soares RA Alviano DS Soares EP Souto-Padron T Lopes AH Rodrigues GC Aguiar AP Pereira MC *Trypanosoma cruzi* peptidases: an overview Open Parasitol J 2010 4 120 131 10.2174/1874421401004010120

[B36] Oliveira-JR Alves CR Silva FS Cortes LMC Toma L Boucas RI Aguilar T Nader HB Pereira MCS *Trypanosoma cruzi* heparin-binding proteins present a flagellar membrane localization and serine proteinase activity Parasitology 2013 140 171 180 10.1017/S0031182012001448 22975090

[B37] Oliveira FO Alves CR Souza-Silva F Calvet CM Côrtes LM Gonzalez MS Toma L Bouças RI Nader HB Pereira MC *Trypanosoma cruzi* heparin-binding proteins mediate the adherence of epimastigotes to the midgut epithelial cells of *Rhodnius prolixus* Parasitology 2012 139 735 743 10.1017/S0031182011002344 22310218

[B38] Carvalho-Moreira CJ Spata MCD Coura JR Garcia ES Azambuja P Gonzalez MS Mello CB *In vivo* and *in vitro* metacyclogenesis tests of two strains of *Trypanosoma cruzi* in the triatomine vectors *Triatoma pseudomaculata* and *Rhodnius neglectus*: short/long-term and comparative study Exp Parasitol 2003 103 102 111 10.1016/S0014-4894(03)00072-9 12880586

[B39] Azambuja P Garcia ES Crampton JM, Beard CB, Louis C Care and maintenance of triatomine colonies Molecular Biology of Insect Disease Vectors: A Methods Manual 1997 London: Chapman and Hall 56 64

[B40] Mello CB Garcia ES Ratcliffe NA Azambuja P *Trypanosoma cruzi* and *Trypanosoma rangeli*: interplay with hemolymph components of *Rhodnius prolixus* J Invertebr Pathol 1995 65 261 268 10.1006/jipa.1995.1040 7745280

[B41] Laemmli UK Cleavage of structural proteins during the assembly of the head of bacteriophage T4 Nature 1970 227 680 685 10.1038/227680a0 5432063

[B42] Rabilloud T Charmont S Rabilloud T Detection of proteins on two-dimensional electrophoresis gels Proteome Research: Two-dimensional Gel Electrophoresis and Identification Methods 2000 New York: Springer 107 126

[B43] Marshall T Silver staining of human salivary proteins following two-dimensional electrophoresis using either protein denaturing or non-denaturing conditions Electrophoresis 1984 5 245 250 10.1002/elps.1150050412

[B44] Lowry OH Rosebrough NJ Farr AL Randall RJ Protein measurement with the folin phenol reagent J Biol Chem 1951 193 265 275 14907713

[B45] Cunha Bastos VL Salles JB Valente RH Leon IR Perales J Dantas RF Albano RM Bastos FF Cunha Bastos J Cytosolic glutathione peroxidase from liver of pacu (*Piaractus mesopotamicus*), a hypoxia-tolerant fish of the Pantanal Biochimie 2007 89 1332 1342 10.1016/j.biochi.2007.04.003 17544198

[B46] Han Y Ma B Zhang K SPIDER: software for protein identification from sequence tags with de novo sequencing error J Bioinform Comput Biol 2005 3 697 716 10.1142/S0219720005001247 16108090

[B47] Han X He L Xin L Shan B Ma B PeaksPTM: Mass spectrometry-based identification of peptides with unspecified modifications J Proteome Res 2011 10 2930 2936 10.1021/pr200153k 21609001

[B48] Ma B Zhang K Hendrie C Liang C Li M PEAKS: powerful software for peptide de novo sequencing by tandem mass spectrometry Rapid Commun Mass Spectrom 2003 17 2337 2342 10.1002/rcm.1196 14558135

[B49] Zhang J Xin L Shan B Chen W Xie M Yuen D Zhang W Zhang Z Lajoie GA Ma B PEAKS DB: de novo sequencing assisted database search for sensitive and accurate peptide identification Mol Cell Proteomics 2012 11 4 M111.010587 doi:10.1074/mcp.M111.010587. Epub 2011 Dec 20 22186715 10.1074/mcp.M111.010587 PMC3322562

[B50] Smith TF Waterman MS Identification of common molecular subsequences J Mol Biol 1981 147 195 197 10.1016/0022-2836(81)90087-5 7265238

[B51] Altschul SF Gish W Miller W Myers EW Lipman DJ Basic local alignment search tool J Mol Biol 1990 215 403 410 2231712 10.1016/S0022-2836(05)80360-2

[B52] Larkin MA Blackshields G Brown NP Chenna R McGettigan PA McWilliam H Valentin F Wallace IM Wilm A Lopez R Thompson JD Gibson TJ Higgins DG Clustal W and Clustal X version 2.0 Bioinformatics 2007 23 2947 2948 10.1093/bioinformatics/btm404 17846036

[B53] Petersen TN Brunak S von Heijne G Nielsen H SignalP 4.0: discriminating signal peptides from transmembrane regions Nat Methods 2011 8 785 786 10.1038/nmeth.1701 21959131

[B54] Whitten M Sun F Tew I Schaub G Soukou C Nappi A Ratcliffe N Differential modulation of *Rhodnius prolixus* nitric oxide activities following challenge with *Trypanosoma rangeli*, *T. cruzi* and bacterial cell wall components Insect Biochem Mol Biol 2007 37 440 452 10.1016/j.ibmb.2007.02.001 17456439

[B55] Paim RM Pereira MH Di Ponzio R Rodrigues JO Guarneri AA Gontijo NF Araújo RN Validation of reference genes for expression analysis in the salivary gland and the intestine of *Rhodnius prolixus* (Hemiptera, Reduviidae) under different experimental conditions by quantitative real-time PCR BMC Res Notes 2012 5 128 10.1186/1756-0500-5-128 22395020 PMC3337225

[B56] Waniek PJ Pacheco Costa JE Jansen AM Costa J Araújo CAC Cathepsin L of *Triatoma brasiliensis* (Reduviidae, Triatominae): sequence characterization, expression pattern and zymography J Insect Physiol 2012 58 178 187 10.1016/j.jinsphys.2011.11.008 22100382

[B57] Abreu PA Albuquerque MG Rodrigues CR Castro HC Structure-function inferences based on molecular modeling, sequence-based methods and biological data analysis of snake venom lectins Toxicon 2006 48 690 701 10.1016/j.toxicon.2006.08.006 17046438

[B58] Guex N Peitsch MC SWISS-MODEL and the Swiss-PdbViewer: an environment for comparative protein modeling Electrophoresis 1997 18 2714 2723 10.1002/elps.1150181505 9504803

[B59] Schwede T Kopp J Guex N Peitsch MC SWISS-MODEL: An automated protein homology-modeling server Nucleic Acids Res 2003 31 3381 3385 10.1093/nar/gkg520 12824332 PMC168927

[B60] Castro HC Silva DM Craik C Zingali RB Structural features of snake thrombin-like enzyme: thrombin and trypsin on a single catalytic platform Biochim Biophys Acta 2001 1547 183 195 10.1016/S0167-4838(01)00177-7 11410274

[B61] Irving JA Pike RN Lesk AM Whisstock J Phylogeny of the serpin superfamily: implications of patterns of amino acid conservation for structure and function Genome Res 2000 10 1845 1864 10.1101/gr.GR-1478R 11116082

[B62] Granzin J Huang Y Topbas C Huang W Wu Z Misra S Hazen SL Blanton RE Lee X Weiergräber O Three-dimensional structure of a schistosome serpin revealing an unusual configuration of the helical subdomain Acta Cryst 2012 D68 686 694 10.1107/S0907444912008372 PMC3370883 22683791

[B63] Noireau F Carbajal-de-la-Fuente AL Lopes CM Diotaiuti CM Some considerations about the ecology of Triatominae An Acad Bras Cienc 2005 77 431 436 16127550 10.1590/s0001-37652005000300006

[B64] Panzera Y Pita S Ferreiro MJ Ferrandis I Lages C Pérez R Silva AE Guerra M Panzera F High dynamics of rDNA cluster location in kissing bug holocentric chromosomes (Triatominae, Heteroptera) Cytogenet Genome Res 2012 138 56 67 10.1159/000341888 22907389

[B65] Pereira MH Souza MEL Vargas AP Martins MS Penido CM Diotaiuti L Anticoagulant activity of *Triatoma infestans* and *Panstrongylus megistus* saliva (Hemiptera/Triatominae) Acta Trop 1996 61 255 261 10.1016/0001-706X(96)00007-1 8790775

[B66] Ribeiro JMC Assumpcao TC Francischetti IMB An insight into the sialomes of bloodsucking Heteroptera Psyche 2012 2012 Article ID 470436, 16 pages doi:10.1155/2012/470436

[B67] Da Mota FF Marinho LP De Carvalho Moreira CJ Lima MM Mello CB Garcia ES Carels N Azambuja P Cultivation-independent methods reveal differences among bacterial gut microbiota in triatomine vectors of Chagas disease PLoS Negl Trop Dis 2012 6 e1631 doi:10.1371/journal.pntd.0001631 10.1371/journal.pntd.0001631 22563511 PMC3341335

[B68] Teixeira ARL Vinaud MC Castro AM Teixeira ARL, Vinaud MC, Castro AM Chagas disease: a global health problem Emerging Chagas Disease 2009 I New York: Bentham Science Publishers 110 121

[B69] Schofield CJ The behaviour of Triatominae (Hemiptera: Reduviidae): a review Bull Entomol Res 1979 69 363 379 10.1017/S0007485300018897

[B70] Hwang WS Weirauch C Evolutionary history of assassin bugs (Insecta: Hemiptera: Reduviidae): Insights from divergence, dating and ancestral state reconstruction PLoS One 2012 7 e45523 doi:10.1371/journal.pone.0045523 10.1371/journal.pone.0045523 23029072 PMC3460966

[B71] Zheng Y-P He W-Y Béliveau C Nisole A Stewart D Zheng SC Doucet D Cusson M Feng QL Cloning, expression and characterization of four serpin-1 cDNA variants from the spruce budworm, *Choristoneura fumiferana* Comp Biochem Physiol B Biochem Mol Biol 2009 154 165 173 10.1016/j.cbpb.2009.05.016 19524698

[B72] Brookhart GL Kramer KJ Proteinases in molting fluid of the tobacco hornworm, *Manduca sexta* Insect Biochem 1990 20 467 477 10.1016/0020-1790(90)90028-S

[B73] Samuels RI Charnley AK Reynolds SE A cuticle degrading proteinase from the moulting fluid of the tobacco hornworm, *Manduca sexta* Insect Biochem Mol Biol 1993 23 607 614 10.1016/0965-1748(93)90034-P 8353521

[B74] Perlowagora-Szumlewicz A Moreira CJC In vivo differentiation of *Trypanosoma cruzi—*1. Experimental evidence of the influence of vector species on metacyclogenesis Mem Inst Oswaldo Cruz 1994 89 603 618 10.1590/S0074-02761994000400018 8524063

[B75] De Stefani Márquez D Rodrigues-Ottaiano C Mônica Oliveira MR Pedrosa AL Cabrine-Santos M Lages-Silva E Ramírezet LE Susceptibility of different triatomine species to *Trypanosoma rangeli* experimental infection Vector Borne Zoonotic Dis 2006 6 50 56 10.1089/vbz.2006.6.50 16584327

[B76] Mello CB Azambuja P Garcia ES Ratcliffe NA Differential in vitro and in vivo behavior of three strains of *Trypanosoma cruzi* in the gut and hemolymph of *Rhodnius prolixus* Exp Parasitol 1996 82 112 121 10.1006/expr.1996.0015 8617337

[B77] Santos CM Jurberg J Galvão C Rocha DS Fernandez JIR Estudo morfométrico do gênero *Panstrongylus* Berg, 1879 (Hemiptera, Reduviidae, Triatominae) Mem Inst Oswaldo Cruz 2003 98 939 944 10.1590/S0074-02762003000700014 14762522

[B78] Barbosa SE Diotaiuti L Braga EM Pereira MH Variability of the salivary proteins of 20 Brazilian populations of *Panstrongylus megistus* (Hemiptera: Reduviidae: Triatominae) Acta Trop 2004 92 25 33 10.1016/j.actatropica.2004.05.012 15301972

[B79] Meiser CK Piechura H Meyer HE Warscheid B Schaub GA Balczun C *A salivary serine protease of the haematophagous reduviid* Panstrongylus megistus*: sequence characterization, expression pattern and characterization of proteolytic activity* Insect Mol Biol 2010 19 409 421 10.1111/j.1365-2583.2010.01002.x 20345395

[B80] Bussacos ACM Nakayasu ES Hecht MM Assumpçao TCF Parente JÁ Soares CMA Santana JM Almeida IC Teixeiraet ARL Redundancy of proteins in the salivary glands of *Panstrongylus megistus* secures prolonged procurement for blood meals J Proteomics 2011 74 1693 1700 10.1016/j.jprot.2011.04.028 21601023

[B81] Meiser CK Piechura H Werner T Dittmeyer-Schafer S Meyer HE Warscheid B Schaub GA Balczun C Kazal-type inhibitors in the stomach of *Panstrongylus megistus* (Triatominae, Reduviidae) Insect Biochem Mol Biol 2010 40 345 353 10.1016/j.ibmb.2010.02.011 20206694

[B82] Tong Y Kanost MR Manduca sexta serpin-4 and serpin-5 inhibit prophenoloxidase activation, cDNA cloning protein expression, and characterization J Biol Chem 2005 280 14923 14931 10.1074/jbc.M500531200 15695807

[B83] Hopkins PC Carrell RW Stone SR Effects of mutations in the hinge region of serpins Biochemistry 1993 32 7650 7657 10.1021/bi00081a008 8347575

[B84] Reichhart J-M Tip of another iceberg: *Drosophila* serpins Trends Cell Biol 2005 15 659 665 10.1016/j.tcb.2005.10.001 16260136

[B85] Schick C Bromme D Bartuski AJ Uemura Y Schechter NM Silverman GA The reactive site loop of the serpin SCCA1 is essential for cysteine proteinase inhibition Proc Natl Acad Sci USA 1998 95 13465 13470 10.1073/pnas.95.23.13465 9811823 PMC24842

[B86] Zhao P Dong Z Duan J Wang G Wang L Li Y Xiang Z Qingyou Xia Q Genome-wide identification and immune response analysis serine protease inhibitor genes in the silkworm, *Bombyx mori* PLoS One 2012 7 e31168 doi:10/1371/journal.pone.0031168 10.1371/journal.pone.0031168 22348050 PMC3278429

[B87] An C Kanost MR *Manduca sexta* serpin-5 regulates prophenoloxidase activation and the toll signaling pathway by inhibiting hemolymph proteinase HP6 Insect Biochem Mol Biol 2010 40 683 689 10.1016/j.ibmb.2010.07.001 20624461 PMC2933306

[B88] Fonseca FPP Ike PTL Assis DM Icimoto MY Juliano MA Juliano L Puzer L Henrique-Silva F Leviserpin: a serin protease inhibitor (serpin) from the sugar cane weevil cane weevil *Sphenophorus levis* Protein J 2011 30 404 412 10.1007/s10930-011-9345-x 21748377

[B89] Zhu Y Wang Y Gorman MJ Jiang H Kanost MR *Manduca sexta* serpin-3 regulates prophenoloxidase activation in response to infection by inhibiting prophenoloxidase-activating proteinases J Biol Chem 2003 278 46556 46564 10.1074/jbc.M309682200 12966082

[B90] Hejgaard J Inhibitory plant serpins with a sequence of three glutamine residues in the reactive center Biol Chem 2005 386 1319 1323 16336127 10.1515/BC.2005.150

[B91] Na Y-R Im H Specific interactions of serpins in their native forms attenuate their conformational transitions Protein Sci 2007 16 1659 1666 10.1110/ps.072838107 17600149 PMC2203359

[B92] Mathialagan N Hansen TR Pepsin-inhibitory activity of the uterine serpins Proc Natl Acad Sci USA 1996 93 3653 13658 10.1073/pnas.93.8.3653 8942989 PMC19381

[B93] De Gregorio E Spellman PT Rubin GM Lemaitre B Genome-wide analysis of the *Drosophila* immune response by using oligonucleotide microarrays Proc Natl Acad Sci USA 2001 98 12590 12595 10.1073/pnas.221458698 11606746 PMC60098

[B94] Castro DP Moraes CS Gonzales MS Ratcliffe NA Azambuja P Garcia ES *Trypanosoma cruzi* immune responses modulation to decrease microbiota in *Rhodnius prolixus* gut is crucial for parasite survival and development PLoS One 2012 7 5 e36591 doi: 10.1371/journal.pone.0036591. Epub 2012 May 4 10.1371/journal.pone.0036591 22574189 PMC3344921

[B95] Gomes SAO Feder D Garcia ES Azambuja P Suppression of prophenoloxidase system in *Rhodnius prolixus* orally infected with *Trypanosoma cruzi* J Insect Physiol 2003 49 829 837 10.1016/S0022-1910(03)00133-1 16256685

[B96] Garcia ES Machado MM Azmabuja P Inhibition of hemocyte microaggregation reactions in *Rhodnius prolixus* larvae orally infected with *Trypanosoma rangeli* Exp Parasitol 2004 107 31 38 10.1016/j.exppara.2004.03.015 15208035

[B97] Whitten MMA Mello CB Gomes SAO Nigam Y Azambuja P Garcia ES Ratcliffe NA Role of superoxide and nitrogen intermediates in *Rhodnius prolixus* (Reduviidae)/*Trypanosoma rangeli* interactions Exp Parasitol 2001 98 44 57 10.1006/expr.2001.4615 11426951

