# Supplementary figures and images for: Isolation and molecular characterization of a major hemolymph serpin from the triatomine, Panstrongylus megistus
Source: Parasit Vectors. 2014 Jan 14;7:23. doi: 10.1186/1756-3305-7-23 (PMC3898217; doi:10.1186/1756-3305-7-23)

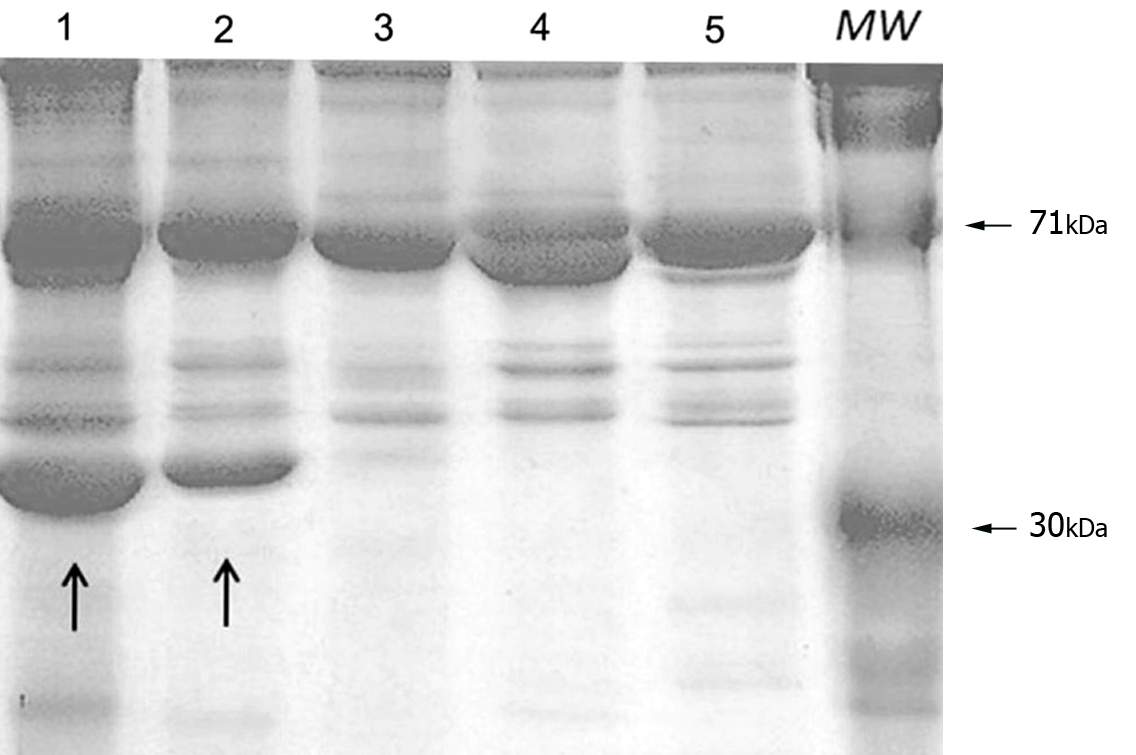

Supplement: Additional file 1: Figure S1 — Hemolymph supernatant protein profile from Panstrongylus lutzi. Samples (0.1 μl of each supernatant) were submitted to 14% SDS-PAGE analysis. Panstrongylus megistus (lane 1), Panstrongylus lutzi (lane 2), Dipetalogaster maximus (lane 3), Rhodnius neglectus (lane 4) and Rhodnius brethesi (lane 5). MW - molecular mass markers. Arrows indicate the target protein at ca. 40 kDa. [file 1756-3305-7-23-S1.tiff]

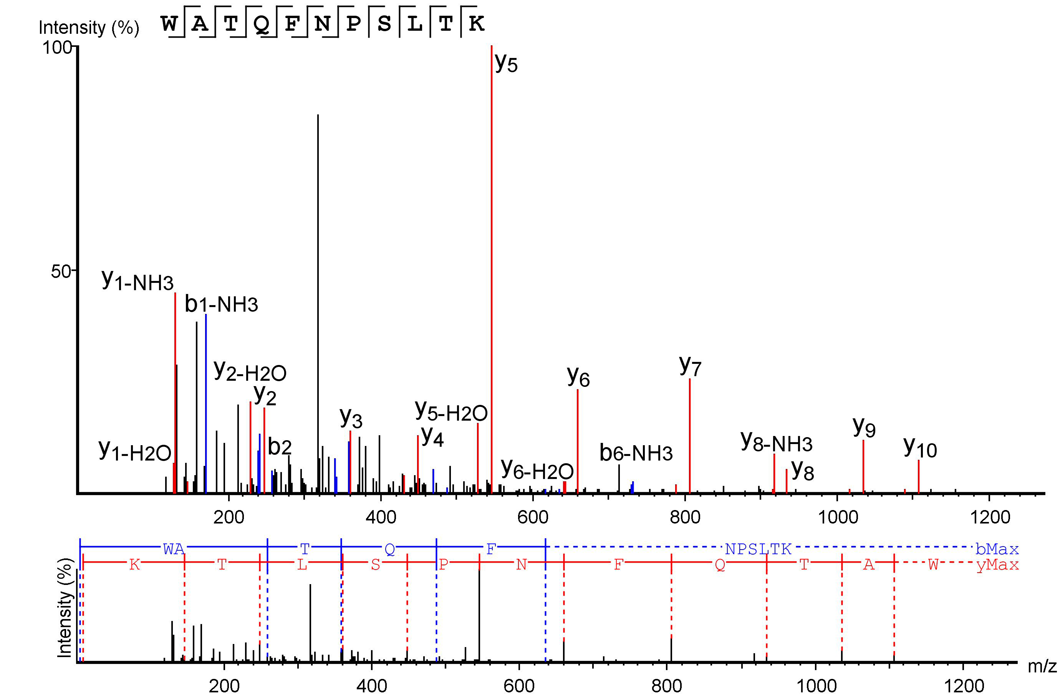

Supplement: Additional file 2: Figure S2 — Fully annotated spectrum for peptide sequence WATQFNPSLTK after de novo sequencing using PEAKS 6.0 software. A precursor mass accuracy of 1.3 ppm and an ALC score of 82% were assigned to this peptide. Data were generated by high resolution acquisitions (Orbitrap analyzer) in MS1 and MS2 modes; fragmentation was performed by HCD (higher energy collisional dissociation) fragmentation. [file 1756-3305-7-23-S2.tiff]

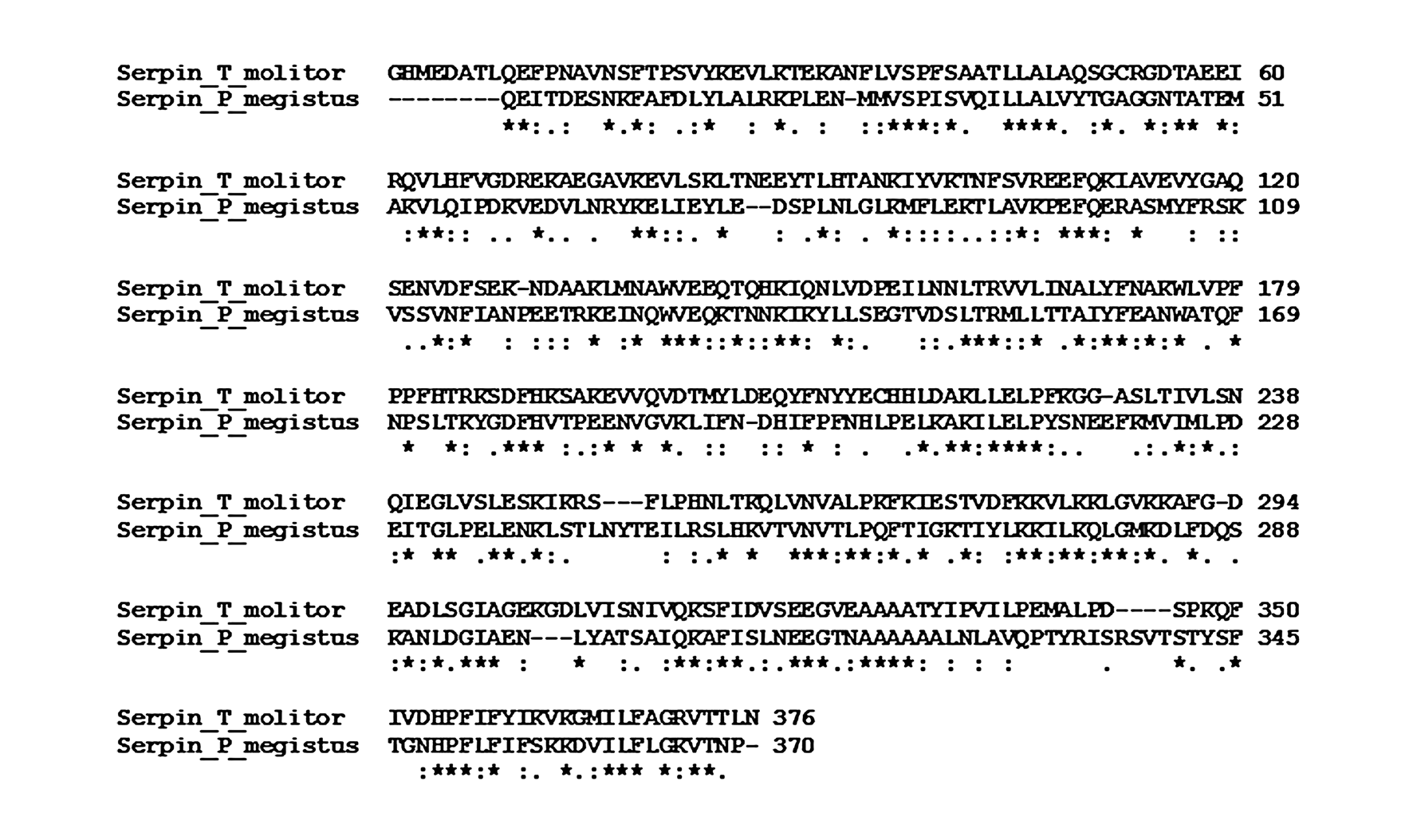

Supplement: Additional file 3: Figure S3 — Alignment of the primary sequence of T. molitor and P. megistus serpins. This alignment was used to construct the 3D model of PMSRP1 shown in Figure 6. The identical amino acids residues are represented by an asterisk (*), whereas residues with similar chemical properties and score > 0.5 in the Gonnet PAM250 matrix are (:) and those with different chemical properties and low-score ≤ 0.5 are (.). [file 1756-3305-7-23-S3.tiff]
